# The interrelation between microbial immunoglobulin coating, vaginal microbiota, ethnicity, and preterm birth

**DOI:** 10.1186/s40168-024-01787-z

**Published:** 2024-05-28

**Authors:** H. J. Schuster, A. C. Breedveld, S. P. F. Matamoros, R. van Eekelen, R. C. Painter, M. Kok, P. J. Hajenius, P. H. M. Savelkoul, M. van Egmond, R. van Houdt

**Affiliations:** 1 Amsterdam UMC location Vrije Universiteit Amsterdam, Medical Microbiology and Infection Control, Boelelaan 1117, Amsterdam, The Netherlands; 2 Amsterdam institute for Immunology and Infectious Diseases, Amsterdam, The Netherlands; 3 Amsterdam UMC location University of Amsterdam, Obstetrics and Gynecology, Meibergdreef 9, Amsterdam, The Netherlands; 4 Amsterdam Reproduction and Development, Amsterdam, The Netherlands; 5 Amsterdam UMC location Vrije Universiteit Amsterdam, Molecular Cell Biology and Immunology, Boeleaan 1117, Amsterdam, The Netherlands; 6 Amsterdam UMC location Vrije Universiteit Amsterdam, Epidemiology and Data Science, Boelelaan 1117, Amsterdam, The Netherlands; 7 Amsterdam UMC location Vrije Universiteit Amsterdam, Obstetrics and Gynaecology, Boelelaan 1117, Amsterdam, The Netherlands; 8 Maastricht University Medical Center+, Medical Microbiology, School of Nutrition and Translational Research in Metabolism (NUTRIM), Maastricht, The Netherlands; 9 Amsterdam UMC location Vrije Universiteit Amsterdam, Surgery, Boelelaan 1117, Amsterdam, The Netherlands

**Keywords:** Vaginal microbiota, Spontaneous preterm birth, Immunoglobulins, Host-microbiota interaction, Ethnicity, Nulliparous women

## Abstract

**Background:**

Vaginal microbiota composition is associated with spontaneous preterm birth (sPTB), depending on ethnicity. Host-microbiota interactions are thought to play an important underlying role in this association between ethnicity, vaginal microbiota and sPTB.

**Methods:**

In a prospective cohort of nulliparous pregnant women, we assessed vaginal microbiota composition, vaginal immunoglobulins (Igs), and local inflammatory markers. We performed a nested case–control study with 19 sPTB cases, matched based on ethnicity and midwifery practice to 19 term controls.

**Results:**

Of the 294 included participants, 23 pregnancies ended in sPTB. We demonstrated that *Lactobacillus iners*-dominated microbiota, diverse microbiota, and ethnicity were all independently associated with sPTB. Microbial Ig coating was associated with both microbiota composition and ethnicity, but a direct association with sPTB was lacking. Microbial IgA and IgG coating were lowest in diverse microbiota, especially in women of any ethnic minority. When correcting for microbiota composition, increased microbial Ig coating correlated with increased inflammation.

**Conclusion:**

In these nulliparous pregnant women, vaginal microbiota composition is strongly associated with sPTB. Our results support that vaginal mucosal Igs might play a pivotal role in microbiota composition, microbiota-related inflammation, and vaginal community disparity within and between ethnicities. This study provides insight in host-microbe interaction, suggesting that vaginal mucosal Igs play an immunomodulatory role similar to that in the intestinal tract.

Video Abstract

**Supplementary Information:**

The online version contains supplementary material available at 10.1186/s40168-024-01787-z.

## Background

An estimated 15 million babies are born preterm each year worldwide [1]. Preterm birth (PTB) is defined as birth before 37 completed weeks of gestation and is a major cause for perinatal mortality and neonatal morbidity [2]. Currently, the prevalence ranges from 5 to 18% across various countries [3]. PTB is usually specified based on its onset, which is either a spontaneous onset of labor (sPTB) or induction of labor or primary caesarean section for maternal or fetal indications [2, 4]. The etiology of sPTB is multifactorial and remains poorly understood. The most important risk factor is a previous sPTB, but most sPTB occur in nulliparous women who lack obstetric history [5]. Several other risk factors have been identified including maternal characteristics such as ethnicity, socio-economic status (SES), body mass index, and maternal smoking, as well as characteristics of the pregnancy like fetal sex, a short mid-trimester cervical length, and intra-amniotic infection [6–9].

Vaginal microbiota play an important role during pregnancy as its composition and dynamics are hypothesized to have an association with sPTB [10–13]. Vaginal microbiota are either of low diversity, mainly dominated by a single *Lactobacillus* species, or consist of a diverse range of (facultative) anaerobic bacteria [14]. Higher diversity of the vaginal microbiota is related to bacterial vaginosis, a disease characterized by increased vaginal discharge and increased susceptibly for invading pathogens [15, 16]. Infection and accompanying inflammatory responses are important risk factors for preterm labor or preterm prelabour rupture of membranes, and about 40% of sPTB is associated with infection [17–19].

Ethnicity is significantly associated with the vaginal microbiota composition. While both low and high diversity vaginal microbiota are found in women of all ethnicities, *Lactobacillus crispatus*-dominant vaginal microbiota is present more often in White European women while diverse vaginal microbiota is present more often in women with a sub-Saharan African descent [20]. The etiology of this association is thus far unknown.

Immunoglobulins (Igs) in the mucosal tissue of the female genital tract are key mediators of mucosal immunology and are important in the defense against infections in the reproductive tract [21]. IgA, the predominant antibody in the intestinal tract, can influence gut microbiota composition [22–24]. Deviations in Ig coating of intestinal bacteria have been associated with inflammatory bowel diseases [25, 26]. In the vaginal tract, there is more IgG than IgA, in contrast to other mucosal surfaces [27]. The etiology of the differences in abundance and type of Igs between mucosal sites is not well understood. In a previous study, our group demonstrated increased microbial IgA coating of *L. crispatus-*dominant vaginal microbiota [28]. Ig coating of vaginal microbiota might play a role in the disparity in microbial community composition between ethnicities and might be associated with gynecological and obstetric diseases.

In this prospective cohort study, we collected vaginal swabs of nulliparous healthy pregnant women at antenatal booking in the first trimester to investigate vaginal microbiota composition and microbial immunoglobulin coating and studied the associations with ethnicity and sPTB. Furthermore, we performed a nested case–control study matching participants with sPTB to participants with uncomplicated term birth. For this subset, we measured unbound Igs and a broad set of inflammatory cytokines and chemokines in vaginal fluid.

## Methods

### Study design and participants

For this study, we used data and vaginal swab material from women included in the PROPELLOR cohort. The study protocol and methods are described in a previous publication [29]. In short, the study included nulliparous women ≥ 18 years who received antenatal care at participating midwifery practices in the Netherlands before 24 weeks of gestation and had a low-risk singleton pregnancy at their first visit. For this study, all participants of whom a vaginal swab and pregnancy outcome was available (no loss to follow-up) were included. Nulliparity was defined as never having had a pregnancy progress beyond 16 weeks of gestation [30]. At the first prenatal visit, usually between 8 and 12 weeks pregnancy, women were approached to participate in this study by their midwife. A self-administered vaginal swab (eSwab, Copan Diagnostics Inc., Murietta, USA) was collected at inclusion. Swabs with the original medium were stored at − 20 °C until transfer at a shipping temperature at − 20 °C to the central storage facility at − 80 °C, storage duration 4–6 years. Written informed consent was obtained from each participant. The study received ethical clearance through the institutional review board of the Academic Medical Center in Amsterdam, the Netherlands (registration number NL43414.018.13).

We analyzed the microbiota composition and microbial bound Igs for all participants. Due to limited funding, we did a nested case control selection of subjects to assess the role of additional inflammatory markers. The additional inflammatory markers included unbound immunoglobulins, cytokines, chemokines, and anti-microbial proteins. Because of financial constraints and missing meta-data, 19 out of 23 women with sPTB were matched based on their ethnicity and midwifery practice to 19 women with term birth. If there was not an appropriate control available within the same midwifery practice, a control was chosen from a midwifery practice in a similar socio-economic region.

### Definitions

The primary outcome measure was sPTB, defined as spontaneous onset of labor or spontaneous preterm prelabor rupture of membranes. We differentiated between sPTB between 23 and 37 weeks of gestation and late second trimester loss between 16 and 22 weeks of gestation. Ethnicity was based on participant self-identification. SES was based on status scores from the Netherlands Institute for Social Research [31].

### Vaginal microbiota and bioinformatics analysis

We pre-treated the vaginal swabs with lysozyme, mutanolysin (Sigma Aldrich, St. Louis, USA), lysostaphin (AMBI, New York, USA), proteinase K, and RNAse A (Thermo Fisher, Waltham, USA). DNA was extracted using the NucliSENS EasyMAG platform, according to manufacturer protocol (BioMérieux, Marcy l’Etoile, France). We used dual indexed universal primers (319F and 806R) for PCR amplification of the V3–V4 regions of the 16S rRNA genes, as described by Fadrosh et al. [32]. PCR products were normalized and pooled. We purified the samples with Agencourt AMPure XP magnetic beads (BeckmanCoulter, Fullerton, USA). Paired-end sequencing was performed on the Illumina MiSeq platform, according to manufacturer protocol (Illumina, San Diego, USA).

We de-multiplexed raw sequences and removed adapters, barcodes, and heterogeneity spacers using Cutadapt 3.5 [33]. Processing of de-multiplexed sequence data and taxonomic classification was performed using the software QIIME 2 version 2021.8 [34]. Forward and reverse reads were trimmed to 260 and 210 basepairs respectively based on a visible drop in average quality beyond these points (visualization performed with https://view.qiime2.org/). We included two unsampled swabs as negative controls. We deemed samples with a read count < 200 reads too similar to the controls and excluded these from analysis. Amplicon sequence variants (ASVs) were generated with DADA2 [35]. We used a pre-trained Naive Bayes classifier of the Silva v138 reference database to assign genus and species (if possible) to each ASV [36]. Because the Silva v138 reference database does not include *Lactobacillus crispatus*, one of the most abundant vaginal species, all *Lactobacillus* sequences without species assignment were further refined using the Nucleotide BLAST (BLASTn) function on the National Center for Biotechnology Information NCBI website. We manually identified sequences belonging to *Candidatus* Lachnocurva vaginae (formerly *Bacterial Vaginosis Associated Bacterium* (BVAB) 1), BVAB2 and TM7-H1. The data were not normalized or rarefied. The code for bioinformatics analyses is available in Supplementary material. Raw sequence reads were dehumanized for publication reasons. We grouped samples based on microbiota profile using VALENCIA centroid classification tool [37]. This tool divides vaginal microbiota into community state types (CSTs) based on their taxonomic composition, by calculating their similarity to a set of reference centroids. It identifies seven CSTS, 4 dominated by lactobacilli (CST I by *L. crispatus*, CST II by *L. gasseri*, CST III by *L. iners*, CST V by *L. jensenii*), and three depleted of lactobacilli (CST IV-A with majority *Candidatus* Lachnocurva vaginae and *Gardnerella vaginalis*, CST IV-B with majority *G. vaginalis* and *Atopobium vaginae*, CST IV-C with low abundance of *G. vaginalis* and *Candidatus* Lachnocurva vaginae). For statistical power, we reduced these to 4 groups, combining CST II and CST V into one group and combining CST IV-A, CST IV-B, and CST IV-C into one group.

### Microbial immunoglobulin coating and inflammatory markers

We determined microbial Ig coating as described in a previous publication using flow cytometry [28]. We calculated coating index by multiplying the percentage of bacteria with bound immunoglobulin with the median fluorescence intensity (MFI). We determined total IgA, IgA1, IgA2, secretory IgA (SIgA), and IgG levels in vaginal swabs by enzyme-linked immunosorbent, as described in a previous publication [28]. We measured vaginal cytokines, chemokines, and anti-microbial peptides with a Multiplex assay using a Bio-Plex 200 according to the manufacturer’s instructions, and human beta defensin-2 (HBD-2) in a separate assay according to manufacturer’s protocol with minor adaptions. We measured total vaginal protein concentration to correct for inter-participant variation according to manufacturer’s protocol. Additional methods and manufacturers can be found in Supplementary methods.

### Statistical analysis

If necessary, data were log-transformed to derive normal distributions. Missing data were handled using multiple imputation creating 10 imputation datasets, except for the main determinants’ vaginal microbiota profile and immunoglobulin coating. All variables, including microbiota composition, microbial bound Igs, and midwifery practice, were used for imputation. Numerical results are based on pooled estimates over 10 imputation sets using Rubin’s rules [38].

For analyses on ethnicity, the variable was dichotomised between White European and non-White European. SES was divided into low and middle/high. We performed Firth’s correction logistic regression analysis for sPTB, calculating ORs and 95% CIs. We defined a priori which associations to estimate and confounders to use and, due to the limited number of events, accounted for the single most important confounder in multivariable logistic regression. Even so, overfitting (overestimations of associations due to small number of events) could be an issue. To reduce overfitting because of the low incidence of sPTB, we applied Firth’s correction for all ORs. Firth’s correction uses penalized likelihood which aims to shrink estimated associations that are overly optimistic [39]. For the association between individual taxa, the linear discriminant analysis effect size (LEfSe) algorithm was used [40]. For this analysis, only bacterial taxa were included with > 1% of total read count. This algorithm calculates the median relative abundance of all taxa and compared this between participants with and without sPTB. It uses factorial Kruskal–Wallis rank-sum test to detect differential abundances of bacterial taxa between these groups. The estimated effect size of the differentially abundant taxa was calculated using linear discriminant analysis, with a minimum threshold of 2.0. For the remaining analyses of the entire cohort, we used Student’s *t*-test and ANOVA with post hoc Bonferroni correction for continuous variables and the Chi-square test for categorical variables. For the nested case–control study, we calculated standardized β-coefficient by linear regression with adjustment for microbiota composition and post hoc Bonferroni correction.

Statistical analyses were performed using IBM SPSS statistics (version 28) and R version 3.3.2 (R Core Team (2016)) with the *mice*, *miceadds*, and *logistf* packages. A *p*-value of < 0.05 was considered statistically significant. Data were visualized using GraphPad Prism (version 9).

### Role of the funding source

The study sponsors had no role in the study design, collection, analysis, and interpretation of the data, the writing of the report, and decision to submit this paper for publication.

## Results

### Study population

A total of 294 participants were included in this study. Key demographics are shown in Table 1. Of the participants, 189 (73.8%) self-identified as White European and 92 (31.3%) had a low SES. sPTB occurred in 23 (7.8%) participants, of which 18 (6.1%) between 23 and 37 weeks of gestation and in five (1.7%) between 16 and 22 weeks of gestation. A miscarriage < 16 weeks of gestation occurred in one (0.3%) participant and in eight (2.7%) participants birth was induced < 37 weeks of gestation for maternal or fetal indications. Key demographics of participants included in the nested case–control study are shown in Table S1.

**Table 1:** Cohort characteristics (N=154). SD: standard deviation. IQR: interquartile range. *including miscarriages < 16+0 weeks of gestation and pregnancy terminations. **including miscarriages < 16+0 weeks of gestation and pregnancy terminations and participants with cervical cerclage or pessary in situ before start routine cervical length measurements

Participant characteristics, total = 294	*n* (%)	Missing
**Maternal age in years at inclusion**, mean (SD)	28.6 (4.1)	0
**Gestational age at inclusion** (weeks), mean (SD)	10.8 (3.4)	0
**Ethnicity**		38 (12.9)
White European	189 (73.8)	
African	26 (10.2)	
Indian	7 (2.7)	
Turkish	3 (1.2)	
Middle Eastern	2 (0.8)	
Asian	4 (1.6)	
Mixed	15 (5.9)	
Other non-Western	10 (3.9)	
**Socio-economic status**		0
Low	92 (31.3)	
Middle	145 (49.3)	
High	57 (19.4)	
**Urinary tract infection during pregnancy** (yes)	21 (7.2)	4 (1.4)
**Vaginal blood loss in 1**^**st**^** or 2**^**nd**^** trimester **(yes)	34 (11.7)	3 (1.0)

### Microbiota

**Fig. 1:**
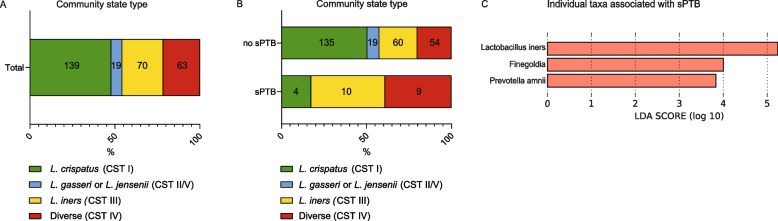
Vaginal microbiota and their association with spontaneous preterm birth (sPTB). **A** Distribution of vaginal community state types (CSTs) of all participants. Numbers represent total participants per group. **B** Distribution of community state types of participants with and without sPTB. Numbers represent total participants per group. **C** The linear discriminant analysis (LDA) score, calculated with linear discriminant analysis effect size (LEfSe) algorithm of the association of individual taxa with sPTB. The bar represents the effect size of the taxa associated with sPTB

Of the 294 samples, three samples were excluded because of a read count below the threshold. The remaining samples had an average of 28,195 reads per sample. 16 s rDNA sequence analysis identified community state types (CSTs), grouped into four clusters: dominated by *L. crispatus* (CST I, *n* = 139), dominated by *L. gasseri* or *L. jensenii* (CST II/V, *n* = 19), dominated by *L. iners* (CST III, *n* = 70), and diverse microbiota (CST IV, *n* = 63) (Fig. 1A and Table S2). *L. iners-*dominated (CST III) and diverse microbiota communities (CST IV) were the most common vaginal microbiota communities found in women experiencing sPTB, and logistic regression revealed an increased odds ratio (OR) for *L. iners*-dominated (CST III) and diverse microbiota (CST IV) compared to women with microbiota dominated by *L. crispatus* (CST I) (OR 5.2, 95% confidence interval (CI) 1.6–16.5 and OR 5.2, 95% CI 1.6–16.9, respectively, Fig. 1B and Table 2). No sPTB occurred in participants with *L. gasseri*/*L. jensenii-*dominated microbiota (CST II/V), resulting in an inaccurate OR. With linear discriminant analysis effect size (LEfSe), the individual taxa *L. iners*, *Finegoldia*, and *Prevotella amnii* were associated with sPTB (Fig. 1C).

**Table 2:** Associations with spontaneous preterm birth. All logistic regression analyses with Firth’ss correction adjusted for gestational age. Only including participants with a pregnancy that progressed beyond 16 weeks of gestation. a Multivariate analyses are adjusted for ethnicity (White European/non-White European), body mass index (continuous) and smoking (no/yes or stopped during pregnancy). Statistically significant data (p<0.05) are presented in bold

Associations with spontaneous preterm birth	OR	95% CI	aOR	95% CI
**Microbiota profile**				
*L. crispatus* (CST I)	ref		ref	
*L. gasseri* or *L. jensenii* (CST II/V)	0.8	< 0.1–16.0	0.7	< 0.1–14.1
*L. iners* (CST III)	**5.2**	**1.6–16.5**	**3.9**	**1.2–12.9**
Diverse (CST IV)	**5.2**	**1.6–16.9**	**3.9**	**1.2–13.2**
**IgA coating index**				
Q1 (< 25%)	ref			
Q2 (25–50%)	0.4	0.1–1.7		
Q3 (50–75%)	1.2	0.4–3.3		
Q4 (> 75%)	0.7	0.2–2.3		
**IgG coating index**				
Q1 (< 25%)	ref			
Q2 (25–50%)	0.8	0.3–2.2		
Q3 (50–75%)	0.6	0.2–1.9		
Q4 (> 75%)	0.5	0.2–1.7		
**Ethnicity**				
White European	ref		ref	
Non-White European	**3.8**	**1.5–9.4**	**2.6**	**1.0–6.5**
**Socio-economic status**				
Middle/high	ref		ref	
Low	**3.8**	**1.6–8.9**	2.4	0.9–6.4
**Urinary tract infection during pregnancy**				
No	ref		ref	
Yes	**4.5**	**1.5–13.4**	**4.0**	**1.3–12.9**
**Vaginal blood loss in 1st or 2nd trimester**				
No	ref		ref	
Yes	**4.0**	**1.5–10.3**	**3.2**	**1.2–8.7**

All logistic regression analyses were performed with Firth’s correction. aOR included at least microbiota profile and ethnicity as factors in the analysis

Statistically significant data (*p* < 0.05) are presented in bold

*OR* odds ratio, *aOR* adjusted OR, *CI* confidence interval, *ref* reference group, *CST* community state type, *Ig* immunoglobulin

### Bacteria bound immunoglobulins

**Fig. 2:**
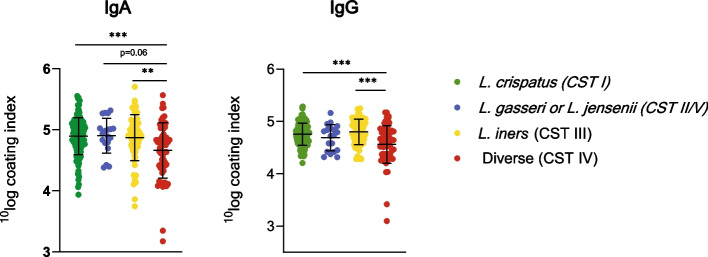
Microbial immunoglobulin coating in different community state types. Bars represent mean with standard deviation. ** *p* < 0.01, *** *p* < 0.001

We measured immunoglobulin coating levels using flow cytometry, calculated coating index of IgA and IgG, and divided these in quartiles. IgA and IgG coating indices were not associated with sPTB (Table 2). However, immunoglobulin coating was associated with microbiota composition (IgA *p* < 0.001, IgG *p* < 0.001) (Fig. 2). IgA and IgG coating index was statistically significantly lower in diverse microbiota (CST IV) compared to *L. crispatus* (CST I) and *L. iners* (CST III)-dominated microbiota (IgA *L. crispatus*/CST I *p* < 0.001 and *L. iners*/CST III *p* = 0.005, IgG *L. crispatus*/CST I *p* < 0.001 and *L. iners*/CST III *p* < 0.001). Two samples with the lowest IgA and IgG coating had both diverse microbiota composition (CST IV), as depicted in Fig. 2. In one sample, *G. vaginalis* was the predominant species and the other was dominated by Enterobacteriaceae. One of the samples with low IgA and IgG coating, with *G. vaginalis* as predominant species, also had lower than average read count (323 reads). Analysis without this sample showed attenuated, but still statistically significant results for IgA coating index (diverse microbiota/CST IV compared to *L. crispatus*/CST I* p* < 0.001 and *L. iners*/CST III* p* = 0.013). For IgG coating, the additional analyses showed the same results as analysis with all participants. We further investigated these results dividing the samples into sub-CSTs (Figure S1, Table S3). Within the group of diverse microbiota, CST IV-A with high to moderate levels of *Candidatus* Lachnocurva vaginae and *G. vaginalis* had IgA and IgG levels similar to *Lactobacillus-*dominated CSTs. CST IV-C2 dominated by *Enterococcus* spp. showed the lowest IgA and IgG coating. Statistical tests were not possible due to small groups.

### Ethnicity

Self-identified ethnicity was associated with sPTB, with an increased risk of sPTB for non-White European women (OR 3.8, 95% CI 1.5–9.4, Table 2). When combining both ethnicity and microbiota composition in a regression model, having *L. iners-*dominated (CST III) or diverse vaginal microbiota (CST IV) and having a non-White European ethnicity remained statistically significantly associated with sPTB, with slightly attenuated adjusted ORs (aORs) (aOR 3.9, 95% CI 1.2–12.9, aOR 3.9, 95% CI 1.2–13.2; and aOR 2.6, 95% CI 1.0–6.5 respectively, Table 2). Several baseline and pregnancy characteristics were associated with sPTB (Table 2). After adjusting for ethnicity and microbiota profile, urinary tract infection during pregnancy and vaginal blood loss in the 1st or 2nd trimester remained associated with sPTB (aOR 4.0, 95% CI 1.3–12.9, and aOR 3.2, 95% CI 1.2–8.7, respectively).

**Fig. 3:**
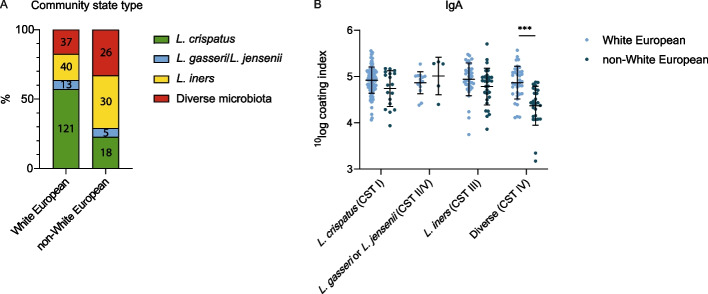
Associations with ethnicity. **A** Distribution of community state types (CSTs) in White European and non-White European. Numbers represent total participants per group. **B** Microbial IgA coating in White European and non-White European participants within various vaginal CSTs. Bars represent mean with standard deviation. *** *p* < 0.001

Ethnicity was also associated with microbiota composition and microbial IgA coating. White European women most often had *L. crispatus-*dominated (CST I) microbiota (*n* = 121, 57.1%) and non-White European women had most often *L. iners-*dominated (CST III) microbiota (*n* = 30, 38.0%) (*p* < 0.001, Fig. 3A). Non-White European participants had lower IgA coating compared to White European women when diverse microbiota was present (*p* < 0.001, Fig. 3B). Analysis without the sample with low read count showed the same results.

### Cytokines, chemokines, and anti-inflammatory peptides

In the nested case–control study, we determined the association between the measured unbound immunoglobulins, cytokines, chemokines, or peptides and sPTB, while adjusting for vaginal microbiota composition (Table S4). None was statistically significantly associated with sPTB. We investigated whether microbial Ig coating and unbound Igs were associated with inflammation. Inflammatory cytokines and chemokines IL-1α, IL-1β, IL-2, IL-6, IL-8, CCL4, and CCL5 were positively associated with one or more of the microbial bound and unbound Igs (Table 3). Also, microbial bound and unbound Igs showed positive correlation (Table S5).

**Table 3:** Correlation between immunoglobulins and inflammatory analytes

	IL-1α	IL-1β	IL-10	IL-13	IL-2	IL-6	IL-8	Eotaxin	CCL4	CCL5	s100A8/A9	HBD-2
Microbial bound IgA	0.531*	0.595***	0.085	0.014	0.319	0.405	0.449*	0.24	0.63***	0.3	− 0.375	0.023
Unbound total IgA	0.398**	0.573***	0.15	0.134	0.533*	0.59**	0.365	0.343	0.705***	0.303	− 0.327	0.089
Unbound IgA1	0.367	0.446	0.142	0.121	0.533*	0.539*	0.221	0.32	0.644***	0.222	− 0.165	0.128
Unbound IgA2	0.456*	0.526**	0.101	0.085	0.400	0.409	0.333	0.276	0.71***	0.31	− 0.427	0.116
Unbound SIgA	0.396*	0.438	0.197	0.107	0.571**	0.419	0.147	0.294	0.611***	0.266	− 0.302	0.062
Microbial bound IgG	0.247	0.398	− 0.127	− 0.059	− 0.030	0.229	0.42	0.045	0.248	0.218	− 0.305	0.043
Unbound IgG	0.326	0.484**	− 0.102	− 0.067	0.166	0.331	0.447	0.108	0.408	0.152	− 0.411	0.201

Correlation expressed as standardized β-coefficient, calculated with linear regression corrected for vaginal microbiota composition. Post hoc Bonferroni correction for multiple comparisons

Statistically significant data (*p* < 0.05) are presented in bold. * corrected *p* < 0.05, ** corrected *p* < 0.01, *** corrected *p* < 0.001

*IL* interleukin, *CCL4* chemokine ligand 4, also known as macrophage inflammatory protein 1β, *CCL5* chemokine ligand 5, also known as RANTES, *s100A8/A9* also known as calprotectin, *HBD* human β-defensin

## Discussion

Our study recapitulated well-known associations between ethnicity and vaginal microbiota composition, with non-White European women having more *L. iners-*dominated (CST III) and diverse microbiota (CST IV), and the association between diverse microbiota and sPTB [11, 14, 41, 42]. The association between *L. iners* and sPTB is previously described, but also an association in the opposite direction with term birth is described [43–45]. Our study adds to the suspicion that *L. iners* is more foe that friend during pregnancy in nulliparous women [46]. Our study describes that diverse microbiota have decreased microbial IgA and IgG coating compared to *Lactobacillus-*dominated microbiota, and that non-White European women with diverse microbiota had lower microbial IgA coating compared to White European women with the same vaginal microbiota profile. With this triad of associations, we anticipated finding an association between decreased IgA coating and sPTB, as diverse microbiota profiles and non-White European participants were over-represented in sPTB cases. The absence of this association was therefore remarkable, and could be reconciled by our finding that lower microbial Ig coating was also associated with lower local inflammation (cytokines and chemokines). Taken together, our findings show that vaginal microbiota and the local immunomodulatory properties of immunoglobulins each play a part in the pathophysiology of preterm birth.

Several studies on sPTB and vaginal microbiota showed similar associations with diverse vaginal microbiota, *L. iners*, and *Prevotella* spp. [10–12, 42, 47–49]. Unique in our study is that the study population only comprises nulliparous women with low risk for sPTB. Parity harbors associations with microbiota composition and sPTB. Vaginal *L. iners* and *Gardnerella* dominance is associated with previous birth and the risk for recurrent sPTB is 30% [50–52]. Therefore, associations between vaginal microbiota and sPTB might be different for nulliparous and multiparous women. This is corroborated by absence of such associations in two recent studies investigating only women at risk for recurrent sPTB [53, 54]. What makes this especially interesting is that the association between vaginal microbiota composition is present in the first trimester. Risk stratification for sPTB early in pregnancy is limited, especially for nulliparous women. Because obstetric history is a strong prognostic factor, prediction models have limited effect in nulliparous women, while this is the largest group at risk [5, 55]. Several treatments can reduce the risk for sPTB, but these are mainly available to women with an increased risk based on obstetric history. Previous studies illustrated the additional value of vaginal microbiota markers to the prediction of sPTB [12, 56, 57]. Our study confirms this, especially identifying a very low risk for sPTB in White European women with *L. crispatus-*dominated (CST I) vaginal microbiota. While this requires further research, determining vaginal microbiota composition in pregnancy could help to provide treatment to nulliparous women with an increased risk for sPTB.

Another strength is that our cohort is large enough to include samples of various vaginal microbiota profiles. This allowed us to further investigate the association between immunoglobulin coating and microbiota profile, compared to our previous study [28]. Also, our study population is ethnically diverse, reflecting the population in large Dutch urban areas and provided us the possibility to investigate ethnic disparities.

A limitation of our study is that despite the size, there were only few sPTB cases, limiting our statistical power. Another limitation is that we did not sample over time and thus longitudinal research was not possible. Also, we found no differences in pro-inflammatory mediators in women with sPTB compared to women delivered at term.

In contrast to recent studies, that showed increased levels of pro-inflammatory mediators in vaginal fluid of women with sPTB, including IL-1β, IL-2, IL-6, IL-8, eotaxin, CCL4, and CCL5 [12, 53, 58, 59]. The most likely explanation for this discrepancy could be the gestational age at collection of the vaginal fluid. In our study, material was collected in early pregnancy (8–12 weeks), while other studies only found statistical differences in samples collected later in pregnancy (> 20 weeks). In one study with multiple sampling moments, no differences were found in samples collected at a first time point between 12 and 16 weeks of gestation, while an increase in several pro-inflammatory mediators between the first and second time point (between 20 and 24 weeks of gestation) was associated with sPTB [53]. These results suggest that inflammatory markers are increasing during pregnancy and clear deviations are not yet found early in pregnancy.

Our study revealed remarkable results concerning microbial bound and unbound Igs. A previous study from our group demonstrated higher IgA coating in *L. crispatus-*dominated vaginal microbiota in non-pregnant healthy women. Due to the larger sample size of the current study, we were able to further elucidate the association between microbial Ig coating. We demonstrated that both IgA and IgG coating are increased in not only *L. crispatus* but all *Lactobacillus* dominated microbiota. The previous longitudinal study focussed on the changes over time and showed higher microbial IgA and IgG coating during menses. As pregnancy is hormonally very different without regular vaginal bleeding, we were interested in the vaginal microbial immunoglobulin coating during pregnancy. Unfortunately, we could not study this longitudinally during pregnancy in the current study. But we did confirm that microbial IgA and IgG coating is also high during pregnancy. Also, the increased the sample size made it possible to study differences in microbial Ig coating in women from different ethnicities.

The association between high microbial coating and high inflammation seems to contradict the association between diverse microbiota and low microbial coating. In previous studies, diverse *Lactobacillus-*depleted microbiota are associated with increased inflammation [12, 53]. Therefore, one would expect to find an association between low microbial coating and high inflammation. However, these results are similar to earlier data from the gut, with IgA both associated with healthy, diversified microbiota, and with inflammatory diseases [25, 60–63]. It has been suggested that low-affinity IgA contributes to healthy gut microbiota, while high-affinity IgA is involved in pathogen clearance [64]. Based on our results, we hypothesize that a similar regulation can take place in the female genital tract. The role of microbial IgG coating remains unclear, as IgG levels are very low in the intestinal tract, and research on vaginal microbial IgG coating especially in relation to *Lactobacillus* spp. is limited. A recent study demonstrated that unbound vaginal IgG levels were highest in diverse microbiota and increasing IgG levels during pregnancy were associated with sPTB [53]. It remains to be elucidated what the exact role of microbial bound and unbound Igs is in vaginal mucosa and genital tract related health outcomes.

Vaginal microbiota and related sPTB risk is associated with ethnicity [10, 11]. Our results imply that ethnicity is also associated with immunoglobulin levels and microbial immunoglobulin binding in the vaginal mucosa. In serum, differential immunoglobulin levels between ethnicity have been identified in studies performed several decades ago. Increased levels of IgG and IgA have been found in Black compared to White populations [65–67]. Also, vaginal cytokine levels in White and Black women have been reported to differ and to be differentially influenced by vaginal microbiota composition [12, 68, 69]. The underlying mechanisms in different immunoglobulin levels and their relation to vaginal microbiota between ethnically diverse women has been understudied and remains open for further investigation both in the circulation and at mucosal surfaces.

## Conclusions

In conclusion, while microbial immunoglobulin coating is associated with vaginal microbiota composition and ethnicity, it is not associated with sPTB. We did find a strong association between *L. iners-*dominated and diverse vaginal microbiota and sPTB in nulliparous women. In addition, we further explored the association between microbial Ig coating and vaginal microbiota composition, showing that diverse vaginal microbiota have lower IgA and IgG coating than *Lactobacillus-*dominated microbiota. Further research should investigate whether microbial immunoglobulin coating plays a role in maintaining a *Lactobacillus-*dominated microbiota profile and whether it is involved in the ethnic disparities of vaginal microbiota composition.

### Supplementary Information

**Supplementary Material 1.** Supplementary methods.

**Supplementary Material 2.** Table S1. Characteristics of participants with short cervix (*n*=136). SD: standard deviation. IQR: interquartile range. *including miscarriage.

**Supplementary Material 3.** Table S2. Read count and community state type per sample. 

**Supplementary Material 4.** Table S3. Sexually transmitted infections. Multiple samples are available per participant and pathogens can be detected in some, but not all, samples from the participant. Therefore, results are presented as sexually transmitted infections (STIs) per participant in total and per sample individually.

**Supplementary Material 5.** Table S4. Associations with spontaneous preterm birth.

**Supplementary Material 6.** Table S5. Correlation between microbial bound and unbound immunoglobulins.

## Data Availability

Due to enhanced privacy legislation regarding the presence of human DNA sequences in publicly available datasets, we cannot make raw sequencing data as used in the analyses in this study publicly available. For publication purposes, human DNA reads were removed from the sequence files using the software HoCoRT and the human genome assembly GRCh38.p14 as reference [70]. As note, the amount of human DNA detected in each file was lower than 1% and should not affect the outcome of any subsequent analysis. The cleaned sequencing data is available under study accession number PRJEB71956, sample accession numbers ERS17760025 to ERS17760318. The read count of individual ASVs per sample is available in Table S2. The data that support the findings of this study, including the raw sequencing data, are available from the corresponding author on reasonable request. Extensive data and material availability is described in a previous publication  [29].
