# Supplementary material for: The interrelation between microbial immunoglobulin coating, vaginal microbiota, ethnicity, and preterm birth
Source: Microbiome. 2024 May 28;12:99. doi: 10.1186/s40168-024-01787-z (PMC11131309; doi:10.1186/s40168-024-01787-z)
Supplement: Supplementary file 2 — Supplementary Material 1. Supplementary methods. [file 40168_2024_1787_MOESM1_ESM.docx]

**Supplementary methods**

**Supplementary methods**

The vaginal swabs were vortexed for 10 seconds to extract bacteria from the swabs. Swab material was centrifuged for 5 min (4000 rpm, 4°C), and blocked with 5% endotoxin free bovine serum albumin (BSA) (Akron Biotech, Boca Raton, FL) for 20 min on ice. Bacteria were stained with F(ab')2 anti-human IgA-AF647 (1:100) and F(ab')2 anti-human IgG-AF488 (1:100) (both Jackson ImmunoResearch, West Grove, USA) for 30 min on ice. Samples were washed in PBS before flow cytometric analysis (LSRFortessa™ X-20, BD Biosciences, Franklin Lakes, USA). For each sample approximately 30.000 events were recorded. Flowcytometric data was visualized and analyzed with FlowJo (version 10.6.2). Coating index was calculated by multiplying the percentage of bacteria with bound immunoglobulin with the median fluorescence intensity (MFI).

**Quantification of unbound immunoglobulins in vaginal fluid**

Total IgA, IgA1, IgA2, secretory IgA (SIgA) and IgG levels in vaginal swabs were determined by enzyme-linked immunosorbent assay (ELISA), as described in Breedveld et al.[1]

**Human beta defensin-2 levels in vaginal fluid**

Human beta defensin-2 (HBD-2) levels in vaginal fluid were measured using the HBD-2 ELISA development kit (cat# LS-F31336, LifeSpan BioSciences, Seattle, USA) following the manufacturer’s instruction with minor adaptations. In brief, 96-well plates (Maxisorb Nalge Nunc) were coated with 50 μL of capture antibody and incubated overnight at room temperature after which the wells were blocked with 150 μL of blocking buffer (0.5% BSA in PBS containing 0.05% Tween20) for 60 min at room temperature. Vaginal swabs were thawed and spun (10.000 rpm, 7 min at 4°C) after which the supernatant was diluted 10 times in blocking buffer and 50 μl was incubated in duplicate for 120 min at room temperature. Wells were incubated with 50 μl detection antibody for 60 min at room temperature after which 50 μl of Avidin-HRP conjugate was added for 30 min. Presence of HBD-2 was detected with 50 μl/well of 12 ml of 0,1M sodium acetate (NaAc pH 4) with 3 μl of 30% hydrogen peroxide and 200 μl of 6 mg/ml 3,3′,5,5′-Tetramethylbenzidine (TMB). The reaction was stopped with 50 μl of sulfuric acid (10% H_2_SO_4_) and absorbance was measured with a microplate reader (Bio-Rad Laboratories, Hercules, USA) at 450 nm.

**Cytokines and chemokines in vaginal fluid**

Cytokine and chemokine levels in vaginal fluid were measured with a Human Custom ProcartaPlex Multiplex 11-plex assay (PPX-11-MXZTEX4, Thermo Fisher Scientific, Waltham, USA) using a Bio-Plex 200 (Bio-Rad, Hercules, California, USA) according to the manufacturer’s instructions. The following mediators were determined in vaginal fluid: Eotaxin (CCL11), interleukin 1 alpha (IL-1α), IL-1β, IL-2, IL-6, IL-8 (CXCL8), IL-10, IL-13, macrophage inflammatory protein 1β (MIP-1β), regulated on activation, normal T cell expressed and secreted (RANTES) and S100A8/A9. Values that were out of range were assigned the upper or lower limit of detection for the specific cytokine. Out of range levels of IL-8 and IL-1β were re-evaluated using their corresponding Ready-SET-Go! ELISA Kit (Sigma Aldrich, Burlington, USA) according to the manufacturer’s instructions.

**Total protein concentration vaginal fluid**

Total protein concentration of every vaginal swab sample was determined using Pierce^TM^ BCA Protein Assay Kits (ThermoFisher, Waltham, USA), according to manufacturer’s protocol. In brief, 10 μL of vaginal fluid (7 times diluted in PBS) was added to a 96-well flat bottom plate, mixed with 200 μL of BCA working reagent and incubated for 30 min at 37°C. Absorbance was measured with a microplate reader (Bio-Rad Laboratories, Hercules, USA) at 562 nm when the plate was cooled to room temperature. To correct for inter participant variation, unbound immunoglobulin and cytokine/chemokine levels (defined with ELISA and Luminex) were corrected for total protein content by dividing the measured mediators by total protein level.

1. Breedveld, A.C., et al., *Enhanced IgA coating of bacteria in women with Lactobacillus crispatus-dominated vaginal microbiota.* Microbiome, 2022. **10**(1): p. 15.
